# High-molecular weight adiponectin/HOMA-IR ratio as a biomarker of metabolic syndrome in urban multiethnic Brazilian subjects

**DOI:** 10.1371/journal.pone.0180947

**Published:** 2017-07-26

**Authors:** Virgínia Genelhu de Abreu, Cyro José de Moraes Martins, Patricia Aguiar Cardoso de Oliveira, Emilio Antonio Francischetti

**Affiliations:** 1 Postgraduate Program on Translational Biomedicine, Grande Rio University, Duque de Caxias, Brazil; 2 Laboratory of Clinical and Experimental Pathophysiology, Rio de Janeiro State University, Rio de Janeiro, Brazil; 3 Pro Rectorate for Research and Postgraduate Programs, Grande Rio University, Duque de Caxias, Brazil; Universita degli Studi Magna Graecia di Catanzaro Scuola di Medicina e Chirurgia, ITALY

## Abstract

Metabolic syndrome (MetS) has an important epidemiological relevance due to its increasing prevalence and association with type 2 diabetes and cardiovascular disease. Insulin resistance is a core feature of the MetS. HOMA-IR is a robust clinical and epidemiological marker of MetS. Adiponectin is an adipokine with insulin-sensitizing and anti-inflammatory functions; its levels decrease as number of components of MetS increases. High-molecular weight adiponectin (HMWA) is the multimer responsible for the relationship of adiponectin with insulin sensitivity. HOMA-IR and HMWA are suitable candidates for MetS biomarkers. The ratio of adiponectin to HOMA-IR has been validated as a powerful index of MetS and considered a better marker of its presence, than either HOMA-IR or adiponectin alone, in selected homogeneous populations. We compared the strength of association between HMWA, HOMA-IR and HMWA/HOMA-IR ratio with MetS and its key components. Our data have shown that the median (25^th^, 75^th^ percentile) of HMWA/HOMA-IR ratio was lower in subjects with MetS [0.51 (0.33, 1.31)] as compared to those without it [2.19 (1.13, 4.71)]. The correlation coefficient (r) was significantly higher for HMWA/HOMA-IR ratio as compared to HMWA for waist circumference (-0.65; -0.40, respectively); mean blood pressure (-0.27; -0.14, respectively); fasting glucose (-0.38; -0.19, respectively); HDL-cholesterol (0.44; 0.40, respectively); and triglycerides (-0.35; -0.18, respectively). In a multivariable logistic regression analysis, the HMWA/HOMA-IR ratio was a sensitive predictor for MetS, being the only marker that was significantly associated with each and all the individual components of the syndrome. These results expand on previous studies in that we used the active circulating form of adiponectin, i.e. HMWA, and represent a typical Brazilian cohort characterized by intense interethnic admixture. Thus, the HMWA/HOMA-IR ratio is a minimally invasive biomarker for MetS that could be clinically useful in prognosing patient outcome.

## Introduction

The metabolic syndrome (MetS), as defined by the Joint Interim Statement of the International Diabetes Federation (IDF), American Heart Association (AHA), National Heart, Lung and Blood Institute (NHLBI) and other international societies [1], has been considered a useful construct because its multiple components cluster with a greater than chance expectation [2]. MetS has also important epidemiological relevance due to its increasing prevalence and association with more life-threatening pathologies, including type-2 diabetes (T2DM) and cardiovascular disease (CVD) [3–6].

There is consistent evidence showing that insulin resistance (IR) is a core feature of MetS; the simultaneous presence of MetS and insulin resistance identifies especially high-risk individuals [6]. The homeostatic model assessment (HOMA-IR) is a mathematical method for assessing IR [7]; it is a robust clinical and epidemiological tool in evaluating IR. Compared with well-validated methods used to measure IR, HOMA-IR and the hyperinsulinemic euglycemic clamp are closely correlated [8].

Adiponectin is a major adipokine with insulin-sensitizing, anti-inflammatory, anti-atherogenic and cardiovascular protective functions [9]. Under metabolically unfavorable conditions, adiponectin is generally downregulated, reduced levels being found in the plasma [10]. Hypoadiponectinemia has been associated with IR [11] and T2DM [12], and with the several components of MetS and with MetS per se [13,14]. Adiponectin also decreases as number of components of MetS increases [14]. In human plasma, adiponectin circulates as oligomeric [high molecular weight (HMW)], hexameric [middle molecular weight (MMW)], and trimeric [low molecular weight (LMW)] forms [15].

It is undoubtedly useful to try characterizing circulating biomarkers for MetS that could be objectively measured in a single blood sample, justifying many efforts that are underway [16,17]. In this regard, HMWA and HOMA-IR are suitable candidates for MetS biomarkers as both largely substantiate our mechanistic understanding of the syndrome, although from different physiologic and pathophysiologic aspects [18,19]. Furthermore, adiponectin and HOMA-IR are independently associated with MetS, although in opposite directions.

The ratio of circulating levels of adiponectin to HOMA-IR (A/H ratio) was seen in 2011 as a powerful index of each component of MetS [20]. Ding et al. [21] noticed that the A/H ratio was a better diagnostic marker of MetS in heathy Chinese subjects than either HOMA-IR or adiponectin alone. These investigators measured total adiponectin, not HMWA; however, it is the quantity of HMWA, not total adiponectin, which is primarily responsible for the relationship of adiponectin with insulin sensitivity, central fat distribution, fat oxidation and lipoprotein subclass profile [22].

The aim of our cross-sectional study has been to compare the strength of association between circulating concentrations of HMWA and HOMA-IR (giving us the HMWA/HOMA-IR ratio) with MetS and its key components. Unlike other reports, we did it in a non-diabetic adult population with varying degrees of adiposity. This cohort is from urban Southeast region of Brazil–metropolitan region of Rio de Janeiro, and represents considerable amount of ethnic admixture. Autosomal Ancestry Informative Markers (AIMs) have shown that this population is significantly differentiated from the ancestral Native American, European and African populations that have been incorporated into its genepool during the last five centuries [23].

Worth mention, the subjects of this report have already participated in previous study conducted by our own group addressing a genetic variant in the endocannabinoid system and its relationship with cardiometabolic risk factors [24].

## Subjects and methods

As described in detail before [24], we analyzed data from 200 subjects, aged 18–60 years (100 eutrophic individuals–body mass index [BMI] ≥18.5 and <25 kg/m2, and 100 obese individuals–BMI ≥30 kg/m2) recruited from the employees, students, residents and staff at the University Hospital, Rio de Janeiro State University. The Research Ethics Committee of Pedro Ernesto University Hospital, Rio de Janeiro State University approved this study without restrictions; subjects voluntarily agreed to participate and provided written informed consent.

The clinical criteria for MetS was defined according to the Joint Interim Statement of the IDF, AHA and NHLBI [1]. The presence of any 3 of 5 risk factors–raised triglycerides, high blood pressure, raised fasting glucose, reduced HDL-Cholesterol and waist circumference ≥90 cm for men and ≥80cm for women—constituted a diagnosis of MetS.

The exclusion criteria were: i) presence of diabetes mellitus or current treatment with hypoglycemic drugs; ii) presence of stage 2 high blood pressure as defined by JNC VII, or current treatment with anti-hypertensive drugs; iii) previous cardiovascular condition (e.g., acute coronary syndrome, cerebrovascular event, or symptomatic peripheral arterial disease); iv) known chronic conditions (e.g., chronic obstructive pulmonary, inflammatory bowel, liver, renal, hematological, psychiatric or autoimmune diseases, endocrinopathies, malignant neoplasms); vi) pregnancy or lactation; vii) herbal or illegal use of drugs; viii) use of drugs for weight loss in the previous 3 months; ix) use of corticosteroids or non-steroidal anti-inflammatory drugs, or use of any drug involved in carbohydrate or lipid metabolism control.

The BMI was determined by dividing the weight in kilograms (kg) by the height in meters squared (m2). Weight was measured using an anthropometric scale (Filizola^™^—Brazil) with a precision of 0.1 kg in fasted subjects who wore light clothes and no shoes. Height was measured using a stadiometer to a precision of 0.5 cm. Waist and hip circumferences were measured with an inelastic tape when the subjects were standing up with the abdomen relaxed and the arms down the sides of the body. The waist circumference (WC) was measured in the middle of the distance between the iliac crest and the last rib. The hip circumference (HC) was measured in the higher posterior circumference of the buttocks. The waist-to-hip ratio (WHR) was obtained by dividing the WC by the HC.

Blood pressure was determined by an oscillatory method using an automatic blood pressure monitor (OMRON, model HEM-705CPINT). The cuff was selected according to the arm circumference. After resting for 5 min, the average of 3 measurements within 3 min was used. The mean blood pressure (MBP) was calculated as the diastolic blood pressure (DBP) plus one-third of the pulse pressure.

Venous blood samples were collected after a 12 h night-fasting period, and the aliquots were stored at -20 or -80°C. Fasting glucose was determined using the enzymatic hexokinase method. Fasting insulin was measured by chemiluminescence. The intra- and inter-assay coefficients of variation were 1.5 and 4.9%, respectively. Insulin resistant status was decided by HOMA-IR [7]. Insulin resistance was defined as a HOMA-IR ≥2.71, according to a threshold value obtained from a multiethnic population in the Brazilian Metabolic Syndrome Study [25]. The lipid profile (triglycerides and total and HDL-cholesterol) was obtained using enzymatic-colorimetric methods. LDL-cholesterol was calculated using Friedewald’s formula [26].

The levels of high sensitive C-reactive protein (hsCRP) were determined by high sensitivity nephelometry. Concentrations of HMWA were measured by ELISA (Millipore Biomanufacturing and Life Science Research–USA). The intra- and inter-assay coefficients of variation were 8.8 and 6.1%, respectively. Leptin was measured with the Milliplex method (Luminex^™^—Human Metabolic Panel, Millipore Corp. USA). The intra- and inter-assay coefficients of variation were 9 and 8%, respectively.

### Statistical analysis

Kolmogorov-Smirnov and Levene tests were used to test the normality of the distribution and homogeneity of variances, respectively. Student’s *t* and Mann-Whitney tests were used for variables with and without a normal distribution, respectively. The partial correlation coefficient was used to analyze correlations among variables with a previous log transformation of the variables without normal distribution and Bonferroni adjustment for multiple testing. To compare the diagnostic strength of the HMWA, HOMA-IR and HMWA/HOMA-IR ratio for classifying subjects with and without MetS, the receiver operating characteristic (ROC) curve analysis was performed and the areas under the curve (AUCs) among HMWA, HOMA-IR, and HMWA/HOMA-IR were compared by a non-parametric approach. Logistic regression analysis was used to determine the odds ratios for the association of MetS and each of its components with the biomarkers. In all statistical analyses, a 2-tailed P value of <0.05 was considered significant. Statistical software PASW Statistics v. 18 (IBM SPSS, Inc.) was used for the analyses.

## Results

The cardiometabolic variables are given (Table 1) in the whole sample, which also compares eutrophic and obese subjects. The levels of HDL-cholesterol, HMWA and the HMWA/HOMA-IR ratio were significantly lower in obese than eutrophic subjects. However, all the other variables were significantly higher in obese subjects, except total cholesterol, which was not significantly different between the groups.

**Table 1:** Clinical and laboratory characteristics of the study population.

Variables	Total Sample (N = 200)M (N = 100) / F (N = 100)	Eutrophics (N = 100)M (N = 50) / F (N = 50)	Obeses (N = 100)M (N = 50) / F (N = 50)	*P* value
**Age (years)**	35.0 ± 10.3	32.4 ± 9.8	37.6 ± 10.2	< 0.001
**Body mass index(kg/m**^**2**^**)**	29.1 ± 7.8	22.5 ± 1.8	35.7 ± 5.5	< 0.001
**Waist circumference (cm)**	94.7 ± 19.3	79.0 ± 7.2	110.4 ± 14.0	< 0.001
**Waist-to-hip ratio**	0.89 ± 0.10	0.84 ± 0.10	0.94 ± 0.08	< 0.001
**Systolic blood pressure (mmHg)**	123.0 ± 14.2	117.4 ± 11.4	128.6 ± 14.6	< 0.001
**Diastolic blood pressure (mmHg)**	75.8 ± 9.7	71.7 ± 7.8	79.9 ± 9.8	< 0.001
**Mean blood pressure (mmHg)**	91.5 ± 10.7	86.9 ± 8.3	96.0 ± 11.0	< 0.001
**Fasting glucose (mmol/L)**	5.02 ± 0.52	4.88 ± 0.43	5.15 ± 0.56	< 0.001
**Total cholesterol (mmol/L)**	4.82 ± 1.05	4.71 ± 0.96	4.93 ± 1.13	> 0.05
**HDL- cholesterol (mmol/L)**	1.36 ± 0.37	1.50 ± 0.35	1.21 ± 0.33	< 0.001
**LDL-cholesterol (mmol/L)**	2.95 ± 0.94	2.79 ± 0.82	3.11 ± 1.01	< 0.05
**Triglycerides (mmol/L)**	0.96 (0.71, 1.38)	0.82 (0.67, 1.01)	1.20 (0.87, 1.68)	< 0.001
**Fasting insulin (pmol/L)**	54.17 (36.11, 90.28)	38.20 (23.61, 52.09)	87.51 (58.34, 111.12)	< 0.001
**HOMA-IR**	1.74 (1.07, 3.00)	1.17 (0.77, 1.64)	2.78 (1.87, 3.60)	< 0.001
**Leptin (ng/ml)**	9.17 (2.79, 19.83)	3.82 (1.35, 9.16)	17.34 (9.07, 30.60)	< 0.001
**HMWA (μg/ml)**	3.04 (1.61, 4.88)	4.11 (2.44, 5.65)	2.20 (1.20, 3.54)	< 0.001
**Hs-C reactive protein (nmol/L)**	18.09 (8.57; 47.62)	10.47 (7.61, 19.04)	34.28 (16.19; 68.57)	< 0.001
**HMWA/HOMA-IR ratio**	1.85 (0.65, 3.88)	3.48 (2.07, 6.52)	0.73 (0.38, 1.65)	< 0.001

Values are means ± standard deviation for variables with normal distribution or median (25^th^ percentile, 75^th^ percentile) for variables without normal distribution.

M, males, F, females, HOMA-IR, homeostatic model assessment of insulin resistance, HMWA, high molecular weight adiponectin, hs-C reactive protein, high sensitive-C reactive protein.

*P* values for differences between eutrophics and obeses (Student *t* test for variables with normal distribution and Mann-Whitney test for variables without normal distribution).

The association between the cardiometabolic variables with the MetS are shown in Table 2. Leptin was the only variable that was not significantly different in subjects with or without MetS. Age, BMI, WC, WHR, blood pressure, glucose, total and LDL-cholesterol, triglycerides, insulin, HOMA-IR and hsCRP were significantly higher in cases with the MetS compared to those without it. Contrariwise, HDL-cholesterol, HMWA and HMWA/HOMA-IR ratio were significantly higher in cases without MetS.

**Table 2:** Cardiometabolic variables in subjects with and without metabolic syndrome.

Variables	Metabolic syndrome		*P* value
	No (N = 155)	Yes (N = 45)	
**Age (years)**	33.5 ± 10.2	39.9 ± 9.0	< 0.001
**Body mass index (kg/m**^**2**^**)**	27.4 ± 7.0	35.1 ± 7.2	< 0.001
**Waist circumference (cm)**	89.8 ± 16.9	111.3 ± 17.5	< 0.001
**Waist-to-hip ratio**	0.87 ± 0.10	0.96 ± 0.07	< 0.001
**Systolic blood pressure (mmHg)**	119.8 ± 12.8	133.8 ± 13.5	< 0.001
**Diastolic blood pressure (mmHg)**	73.9 ± 8.9	82.3 ± 9.4	< 0.001
**Mean blood pressure (mmHg)**	89.1 ± 9.71	99.4 ± 10.3	< 0.001
**Fasting glucose (mmol/L)**	4.91 ± 0.44	5.41 ± 0.59	< 0.001
**Total cholesterol (mmol/L)**	4.73 ± 1.01	5.17 ± 1.13	0.01
**HDL-cholesterol (mmol/L)**	1.45 ± 0.36	1.06 ± 0.22	< 0.001
**LDL-cholesterol (mmol/L)**	2.85 ± 0.90	3.30 ± 1.00	0.004
**Triglycerides (mmol/L)**	0.86 (0.68, 1.15)	1.69 (1.21, 2.17)	< 0.001
**Fasting insulin (pmol/L)**	50.70 (29.17, 79.17)	84.73 (57.64, 104.87)	< 0.001
**HOMA-IR**	1.61 (0.91, 2.38)	2.99 (1.94, 3.91)	< 0.001
**Leptin (ng/mL)**	7.89 (2.26, 20.16)	12.41 (4.29, 18.29)	0.06
**HMWA (μg/mL)**	3.55 (2.05, 5.25)	1.64 (1.12, 2.56)	< 0.001
**High-sensitive C reactive protein (nmol/L)**	16.9 (8.57, 43.81)	28.57 (14.29, 61.91)	0.01
**HMWA/HOMA-IR Ratio**	2.19 (1.13, 4.71)	0.51 (0.33, 1.31)	< 0.001

Values are mean ± standard deviation for variables with normal distribution or median (25^th^ percentile, 75^th^ percentile) for variables without normal distribution.

*P* value for the difference between variables in subjects with and without metabolic syndrome (Student *t* test for variables with normal distribution and Mann-Whitney test for variables without normal distribution).

HOMA-IR, homeostatic model assessment of insulin resistance; HMWA, high molecular weight adiponectin.

The partial correlations between the cardiometabolic risk factors with HMWA, HOMA-IR and HMWA/HOMA-IR ratio, adjusted for age and gender, are given in Table 3. Concerning the variables that are components of the MetS–WC, blood pressure, glucose and triglycerides—these were negatively correlated with HMWA and the HMWA/HOMA-IR ratio. The magnitude of the effect (correlation coefficient) was higher for HMWA/HOMA-IR ratio compared to HMWA. Contrariwise, HDL-cholesterol was positively correlated with HMWA and the HMWA/HOMA-IR ratio. For HOMA-IR, positive correlations were WC, blood pressure, glucose and triglycerides, whereas HDL-cholesterol was negatively correlated.

**Table 3:** Partial correlations* between HMWA, HOMA-IR and the HMWA/HOMA-IR ratio with cardiometabolic variables.

Variables	HMWA		HOMA-IR		HMWA/HOMA-IR Ratio	
	r	*P*	r	*P*	r	*P*
**Body mass index**	-0.36	<0.001	0.65	<0.001	-0.58	<0.001
**Waist circumference**^a^	-0.40	<0.001	0.70	<0.001	-0.65	<0.001
**Waist-to-hip Ratio**	-0.27	<0.001	0.47	<0.001	-0.40	<0.001
**Systolic blood pressure**^a^	-0.14	0.03	0.25	<0.001	-0.24	0.001
**Diastolic blood pressure**^a^	-0.13	0.05	0.30	<0.001	-0.26	<0.001
**Mean blood pressure**	-0.14	0.03	0.31	<0.001	-0.27	<0.001
**Fasting glucose**^a^	-0.19	0.007	0.39	<0.001	-0.38	<0.001
**Total cholesterol**	0.07	0.29	0.14	0.04	-.004	0.95
**HDL-cholesterol**^a^	0.40	<0.001	-0.34	<0.001	0.44	<0.001
**LDL-cholesterol**	-0.01	0.86	0.18	0.01	-0.07	0.31
**Triglycerides**^a^ ^b^	-0.18	0.01	0.40	<0.001	-0.35	<0.001
**Fasting insulin**^b^	-0.36	<0.001	0.98	<0.001	-0.81	<0.001
**HOMA-IR**^b^	-0.37	<0.001	-	-	-0.83	<0.001
**Leptin**^b^	-0.26	<0.001	0.66	<0.001	-0.56	<0.001
**HMWA**^b^	-	-	-0.42	<0.001	0.85	<0.001
**High-sensitive C-reactive protein**^b^	-0.16	0.02	0.28	<0.001	-0.24	0.001
**HMWA/HOMA-IR ratio**^b^	0.85	<0.001	-0.83	<0.001	-	-

*Adjusted for age and gender.

^a^Components of metabolic syndrome definition.

^b^Variables log-transformed before analysis.

HMWA, high molecular weight adiponectin; HOMA-IR, homeostatic model assessment of insulin resistance.

To address the association between the MetS with HMWA, HOMA-IR and HMWA/HOMA-IR ratio, multivariable logistic regression was used, with MetS as the dichotomous outcome variable and the quartiles of HMWA, HOMA-IR and HMWA/HOMA-IR ratio as the predictor variables, based on 2 models (Table 4). In Model 1, the control variables were age and gender, whereas Model 2 controlled for age, gender, BMI and total cholesterol. The adjusted odds ratios for MetS were higher with the HMWA/HOMA-IR ratio than with HMWA or HOMA-IR, and remained significantly associated along all the quartiles.

**Table 4:** Odds ratios (95% CI) for associations between MetS and HMWA, HOMA-IR and HMWA/HOMA-IR ratio.

Quartile	Model 1	Model 2
**HMWA**		
**First**	1	1
**Second**	0.44 (0.18–1.06)	0.48 (0.18–1.27)
**Third**	0.11 (0.03–0.38)	0.14 (0.04–0.52)
**Fourth**	0.07 (0.02–0.29)	0.10 (0.02–0.50)
**HOMA—IR**		
**First**	1	1
**Second**	0.70 (0.18–2.67)	0.41 (0.10–1.72)
**Third**	2.50 (0.83–7.53)	0.93 (0.26–3.35)
**Fourth**	5.00 (1.81–13.8)	1.30 (0.35–4.79)
**HMWA/HOMA—IR RATIO**		
**First**	1	1
**Second**	0.15 (0.05–0.42)	0.18 (0.06–0.51)
**Third**	0.18 (0.06–0.49)	0.30 (0.09–0.92)
**Fourth**	0.03 (0.01–0.15	0.09 (0.01–0.50)

Model 1 –Adjusted for age and gender

Model 2—Adjusted for age, gender, body mass index and total cholesterol

MetS, metabolic syndrome; 95% CI, 95% confidence interval; HMWA, high molecular weight adiponectin; HOMA-IR, homeostatic model assessment of insulin resistance.

**Fig 1:**
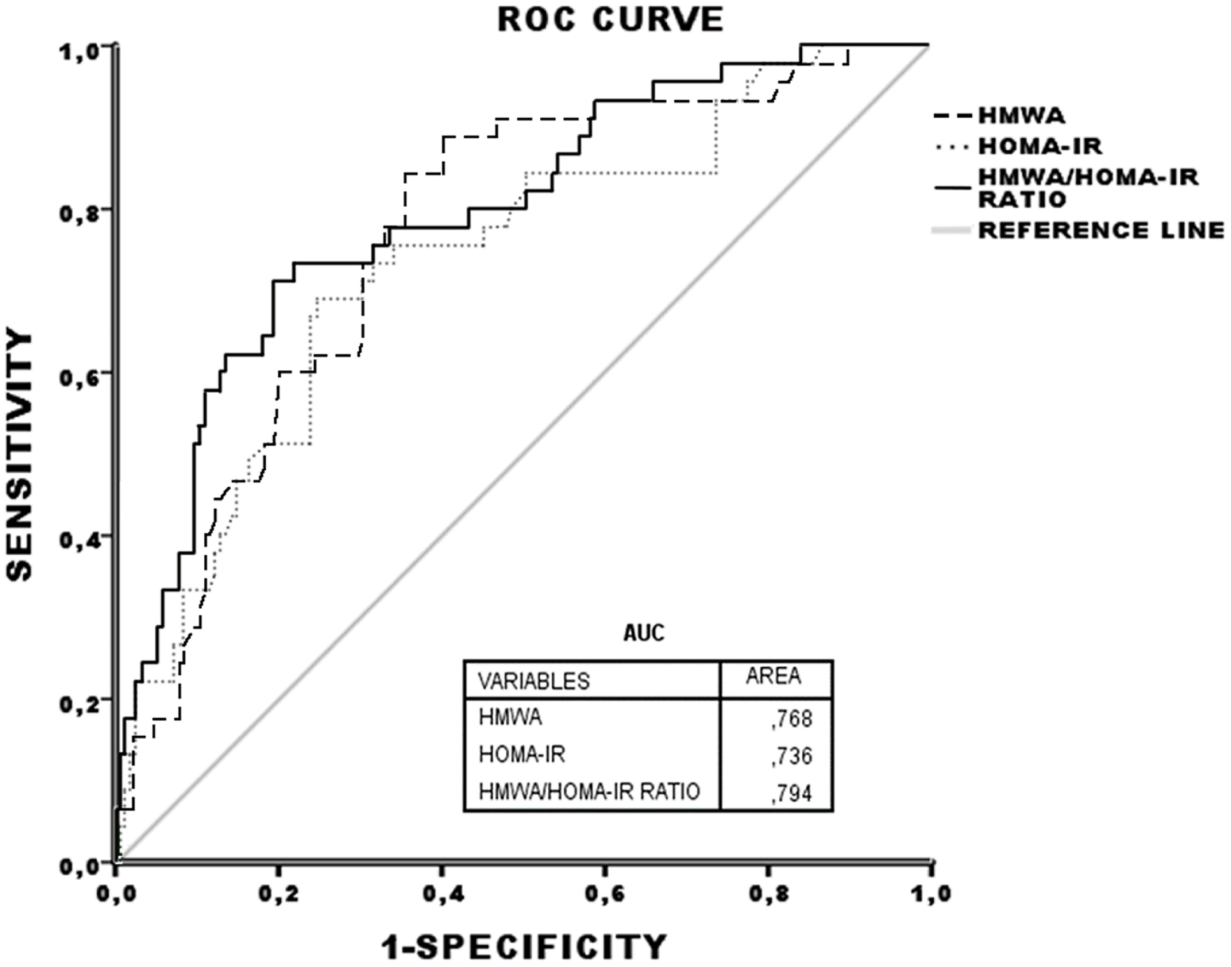
Comparison of predicting powers between HMWA, HOMA-IR and HMWA/HOMA-IR ratio for metabolic syndrome. HMWA, high molecular weight adiponectin; HOMA-IR, homeostatic model assessment of insulin resistance; ROC, receiver operating characteristic; AUC, area under the curve.

To compare the predictive powers of HMWA, HOMA-IR and the HMWA/HOMA-IR ratio for the MetS, ROC curves were plotted (Fig 1). The area under the curve (AUC) was greater for the HMWA/HOMA-IR ratio compared to HMWA and HOMA-IR.

In further analysis, the association of each MetS component with HMWA, HOMA-IR and the HMWA/HOMA-IR ratio, the adjusted odds ratios between the highest and lowest quartiles and the ROC curves analyses were determined. HMWA/HOMA-IR ratio was the only marker significantly associated with each and all the individual components of MetS (Table 5).

**Table 5:** Odds ratios* (95% CI) and ROC curves analysis for the association between each component of MetS and HMWA, HOMA-IR and HMWA/HOMA-IR ratio.

	HMWA(Q4 x Q1)		HOMA-IR (Q4 X Q1)		HMWA/HOMA-IR Ratio (Q4 X Q1)	
	OR (95% CI)	AUC (SE)	OR (95% CI)	AUC (SE)	OR (95% CI)	AUC (SE)
**Abdominal Obesity**	0.10 (0.04–0.28)	0.66 (0.04)	43.08 (12.20–152.05)	0.80 (0.03)	0.01 (0.01–0.06)	0.81 (0.03)
**High Triglycerides**	0.08 (0.01–0.64)	0.71 (0.05)	1.68 (0.49–5.80)	0.57 (0.06)	0.13 (0.03–0.63)	0.68 (0.05)
**High Blood Pressure**	0.50 (0.20–1.23)	0.61 (0.04)	2.48 (1.04–5.91)	0.63 (0.05)	0.21 (0.08–0.54)	0.67 (0.04)
**Low HDL-cholesterol**	0.08 (0.03–0.27)	0.69 (0.04)	6.33 (2.35–17.05)	0.69 (0.04)	0.07 (0.02–0.21)	0.75 (0.04)
**High Fasting Glucose**	0.69 (0.24–1.96)	0.59 (0.06)	9.71 (2.04–46.20)	0.69 (0.05)	0.17 (0.05–0.57)	0.70 (0.05)

*Adjusted for age and gender.

95% CI, 95% confidence interval; ROC, receiver operating characteristic curve; MetS, metabolic syndrome; HMWA, high molecular weight adiponectin; HOMA–IR, homeostatic model assessment of insulin resistance; OR, odds ratios; AUC, area under the curve; SE, standard error; Q4, highest quartile; Q1, lowest quartile.

## Discussion

MetS is a serious global issue with substantial clinical relevance and increasing prevalence. Data from the National Health and Nutrition Examination Survey (NHANES, 1999–2002) showed that 34.5% of Americans met the criteria for MetS compared with 22% in NHANES III, 1988–1994 [4,5]. Similarly, the risks of incident T2DM and CVD increased 4.6 and 1.7 fold in subjects with MetS compared with subjects without MetS [6]. We found the HMWA/HOMA-IR was more strongly associated with MetS than HMWA, and gave a better predictive power for MetS—controlled for age, gender, BMI and total cholesterol, when compared with HMWA and HOMA-IR alone. Furthermore, only the HMWA/HOMA-IR ratio showed any significant relationship with each and all the individual components of MetS.

There are few reports on the adiponectin/HOMA-IR ratio as an index of MetS. In a community-based study of an aged Japanese population, Nakatoshi et al [20] found that the total adiponectin/HOMA-IR (A/H) ratio was a powerful index of the components of MetS, each component of MetS, or the absence of MetS. They suggested that the measurement of adiponectin plasma levels, insulin, fasting plasma glucose and HOMA-IR provided the basis for a screening test to confirm or exclude MetS. Ding et al [21] assessed 1628 healthy Chinese subjects regarding the diagnostic efficacy of adiponectin, HOMA-IR and the A/H ratio to identify MetS and MetS components. Association of these biomarkers with MetS and its components using logistic regression analysis and ROC curves showed that the A/H ratio was a more robust marker for the diagnosis of MetS than either HOMA-IR and adiponectin alone. A study of obese Spanish women showed that the A/H ratio provided a discriminatory marker for MetS risk and some components of MetS (central obesity, increased blood pressure and elevated fasting plasma glucose). However, there was an important limitation related to age, since older women (mean age 54.3 years) attending the criteria for MetS (and probably in the post-menopause period) were compared with younger women (mean age 41.9 years) without MetS [27].

What remains unknown is whether the HMWA/HOMA-IR ratio per se has additional significance for the early characterization of MetS beyond that of HMWA and HOMA-IR. Although the A/H ratio is a recognized index for the risk of MetS, HMWA is responsible for these relationships [28]. Moreover, other reports have shown consistent evidence that HMWA is the active form of adiponectin. For example, HMWA was shown to stimulate phosphorylation of 5`- adenosine monophosphate—activated protein kinase (one of the key molecules mediating the metabolic actions of adiponectin) [29]—besides being the most active form suppressing hepatic glucose production [30]. Additionally, the anti-atherogenic properties of adiponectin seem to be mediated preferentially by HMWA through effects that selectively suppress endothelial cell apoptosis and promote a more favorable lipoprotein subclass profile [31].

In their seminal study, Lara-Castro et al [22] examined the relationship between circulating levels of total adiponectin, adiponectin multimers (LMWA and HMWA) and the HMWA-to-total adiponectin ratio with the key features of MetS. They elegantly showed that HMWA (not total or HMWA-to-total adiponectin or LMWA) was responsible for all these relationships, which also included mechanistic features of MetS, such as central fat distribution, increasing ratio of fat oxidation, insulin-stimulated glucose disposal rate, and lipoprotein subclasses profile associated with insulin resistance.

Noteworthily, these studies were on Asian and European Caucasian cohorts. Thus, they do not reflect the significant racial-ethnic differences in circulating adipokines, as has been clearly demonstrated in the Study of Women’s Health Across the Nation (SWAN) carried out in the United States [32]. To the best of our knowledge, our data provide the novel data on the relationship of the HMWA/HOMA-IR ratio with the MetS trait cluster in a multi-ethnic population. This is a new approach because it might allow the extension of these results to other multi-ethnic and intensive interbreeding populations of South and Central America.

There are strengths and limitations regarding the results of our study. As the individuals included in our analysis were apparently heathy subjects, they had not been using any drugs that might interfere in carbohydrate and lipid metabolism, such as anti-hypertensive and lipid lowering agents. The distribution and quantification of central fat mass were measured by indirect methods and the insulin resistance was calculated by HOMA-IR. Although these methods are surrogates outcomes, they have generally been validated by several population studies.

Major limitations of our study were the small size of the cohort and its nonprobabilistic sampling method, which inevitably would limit its predictive power, although it has the advantage of using a multiethnic population. In addition, our study was cross-sectional in nature and, hence, not designed to establish a cause-and-effect relationship, but rather associations.

## Conclusions

As a consequence of the epidemic of obesity, prediction of MetS is relevant because of its subsequent association with T2DM and CVD. Regarding the variables that are components of the MetS–waist circumference, blood pressure, glucose, HDL-cholesterol and triglycerides—our data show that the HMWA/HOMA-IR ratio is a sensitive predictor for MetS, being the only marker that was significantly associated with each and all the individual components of the syndrome. Thus, the HMWA/HOMA-IR ratio is a circulating and minimally invasive biomarker for MetS that could be clinically useful in prognosing patient outcome. These results expand on previous studies since they represent a typical Brazilian cohort characterized by intense interethnic admixture.

## Supporting information

S1 Datafile This is the research databank. (XLS)
